# Supplementary material for: Perfusion of MC3T3E1 Preosteoblast Spheroids within Polysaccharide-Based Hydrogel Scaffolds: An Experimental and Numerical Study at the Bioreactor Scale
Source: Bioengineering (Basel). 2023 Jul 18;10(7):849. doi: 10.3390/bioengineering10070849 (PMC10376891; doi:10.3390/bioengineering10070849)
Supplement: Supplementary file 1 [file bioengineering-10-00849-s001.zip › bioengineering-2452024-supplementary.pdf]

bioengineering

Supplementary Information

Perfusion of MC3T3E1 Preosteoblast Spheroids within Polysaccharide-Based Hydrogel Scaffolds: An Experimental and Numerical Study at the Bioreactor Scale

Jérôme Grenier [1,2,3], Bertrand David [2,*], Clément Journé [3], Iwona Cicha [4], Didier Letourneur [3,†] and Hervé Duval [1,†]

S1 – Cell labeling with superparamagnetic iron oxide nanoparticles
S2 – MRI sequences settings
S3 – Method for the assessment of cell number using T2*-MSME relaxometry
S4 – Unsteady diffusion model to identify the oxygen diffusion coefficient
S5 – Lattice-Boltzmann Method for oxygen transport simulation
S6 – Cumulative volume-average distribution of spheroid diameters
S7 – Mass transfer limitations according to standard correlations

Supplementary Material 1: Cell labeling with superparamagnetic iron oxide nanoparticles

MC3T3-E1 osteoblasts were labeled with biocompatible superparamagnetic iron oxide nanoparticles (SPION^{LA-HSA}) that were synthesized according to Zaloga et al. [27] by iron II and iron III salts precipitation, and coated with lauric acid (LA) and human serum albumin (HSA). The superparamagnetic core size was 20 to 30 nm, and the hydrodynamic diameter was measured by dynamic light scattering (DLS) at 74.44 nm. The organic layer of coating was shown to stabilize the nanoparticles and increase their biocompatibility up to 200 $\mu g_{[Fe]}$ cm^{-2} [27].

For labeling, MC3T3E1 osteoblasts were seeded on a culture plate (7,500 cells per cm^2). At 24 h post-seeding, the culture medium was supplemented with SPION^{LA-HSA} (20 $\mu g_{[Fe]}$ cm^{-2}), and cells were incubated for 36 h. Then, the culture medium was refreshed for the remaining 12 h before cell harvesting.

Supplementary Material 2: MRI sequences settings

The 7 Tesla MRI (Pharmascan 70/16, Bruker, Hôpital Bichat, Paris) controlled by ParaVision® software was used with a 40 mm body mouse volume antenna that was calibrated in frequency.

The "T2-turbo RARE" sequence was used with rare factor = 8 and 2 images averaging. The acquisition matrix consisted of 135 axial slices of 250 µm thickness and 55 µm resolution in the Z-direction of the PP tube. Echo time T_E = 60 ms and repetition time T_R = 17,285 ms. The acquisition was duplicated after translation of 125 µm in the Z-direction. The two acquisitions lasted 1h and 10 minutes. 3D reconstruction was performed with Fiji (ImageJ) to achieve a 125 µm definition in the Z-direction by alternation of the slices. Polynomial interpolation was performed to create a 3D numerical model of the bioreactor with 55 µm resolution. 3D image processing that included non-local mean filtering, thresholding, and segmentation allowed us to define four phases: 1) the fluid, 2) the hydrogel scaffolds, 3) the cells, and 4) outside limits of the tubes.

The "T2*-MSME (Multi-Slice-Mutli-Echo)" sequence was used to quantify by relaxometry the concentration of SPION^{LA-HSA}. The acquisition matrix consisted of a single sagittal slice of 250 µm thickness and 55 µm resolution parallel to the Z-direction of the tubes. Minimal echo time T_E = 13.845 ms with 11 spacing and repetition time T_R = 2,000 ms. The acquisition lasted 1 h with four images averaging. T_2^* coefficient was estimated by fitting the exponential decrease of the signal S with the equation proposed by Milford et al. [33]:

$$S(T_E) = \text{offset} + k \cdot S_0 \cdot \exp\left(\frac{-T_E}{T_2^*}\right) \quad \text{(S1)}$$

with S_0 the proton density, k, a proportional coefficient, and offset, a constant introduced by Milford et al. [33] that was computed from the averaging of the three last signals.

Supplementary Material 3: Assessment of cell number using T2*-MSME relaxometry

Using the T2-turbo RARE sequence allowed a successful correlation of the number of cells seeded within the porous hydrogel scaffolds. However, the estimated number of cells was 10 times higher than the theoretical number of seeded cells. This may be due to an overestimation of the size of the cell clusters, whose average diameters were lower than the MRI resolution. In an attempt to overcome this limitation, analysis of the cellularized scaffold hydrogel was carried out by MRI relaxometry using the T_2^*-MSME sequence (**Figure 5 A**). The average relaxation coefficient T_2^* was 30.4 ms ($R^2 = 0.976$) for scaffolds seeded with 300,000 cells (**Figure 5 A1**), and 30.3 ms ($R^2 = 0.983$) in those seeded with 600,000 cells (**Figure 5 A2**). This result indicated that there was no correlation between T_2^* and the seeded cell number under these conditions. The average relaxation coefficient T_2^* was 93.6 ms for dark voxels (hyposignal) corresponding to cell clusters with a poor description of the data by the model ($R^2 = 0.534$) (**Figure 5 A3**). This result indicated that echo times T_E were not small enough to capture the relaxation signal generated by the cells [45]. To improve this method [45], one may thus want to optimize the minimum echo time to a smaller time than the 13.845 ms used in this study, as well as to optimize/reduce the $SPION^{LA\text{-}HSA}$ concentration below 20 $\mu g_{[Fe]}$ cm^{-2} used here.

Supplementary Material 4: Unsteady diffusion model to identify the oxygen diffusion coefficient

The unsteady diffusion model proposed by Carman et Haul [35] was used to analyze the experimental oxygen concentration data. The hydrogel was considered as a solid media with thickness e that exchanges oxygen through one side, with the finite well-stirred volume inside the main chamber. The other sides were assumed to be insulated. Hydrogel volume was $V_h = \pi e(d/2)^2$. Dissolved oxygen concentration $c(t)$ follows the simplified expression:

$$\ln\left(\frac{c(t) - c_\infty}{c_2 - c_\infty}\right) \cong \ln\left(\frac{2(1+\Lambda)}{1+\Lambda+q_1^2\Lambda^2}\right) - \frac{q_1^2 Dt}{e^2} \quad \text{(S2)}$$

with D, the oxygen diffusion coefficient, Λ, the distribution coefficient that is computed as

$$\Lambda = \frac{c_\infty - c_1}{c_2 - c_\infty} \quad \text{(S3)}$$

and q_1, the first non-zero root of the equation:

$$\tan q + \Lambda q = 0 \quad \text{(S4)}$$

At equilibrium, the mass conservation gives:

$$V_r\,(c_2 - c_\infty) = V_h(K c_\infty - K\,c_1) \quad \text{(S5)}$$

where K is the oxygen partition coefficient between the hydrogel and the PBS.
Therefore, K is given by :

$$K = \frac{V_r}{V_h}\frac{1}{\Lambda} \quad \text{(S6)}$$

Underlying assumptions for equation **Error! Reference source not found.** are: (i) $\Lambda > 0.4$, i.e., the volume ratio V_r/V_h is high enough and (ii) $(c(t) - c_\infty)/(c_2 - c_\infty) < 0.45$, i.e., the time t is long enough.

In our experimental results, the concentration step was characterized by the distribution factor $\Lambda = 3.05 > 0.4$, which satisfied hypothesis (i). For $c(t) < 3.63$ mg.L^{-1}, hypothesis (ii) was satisfied, and D was determined from the slope of the linear regression ($R^2 = 0.985$) of $-\ln(c(t) - c_\infty)$ against time, following equation **Error! Reference source not found.**.

Supplementary Material 5: Lattice Boltzmann Method for oxygen transport simulation

Two-Relaxation-Time Lattice Boltzmann Method (TRT LBM) with a cubic lattice in three dimensions and seven velocities (D3Q7) was used to describe the oxygen transport [44].

As for the simulations of the fluid flow (see Section 2.7.1), the oxygen concentration within the bioreactor was described by fictitious particles populations f_q distributed on the nodes $\boldsymbol{r}$ of a cubic lattice. The three-dimensional velocity set (D3Q7) includes one zero vector $\boldsymbol{c}_0$ and $(Q-1)=6$ non-zero vectors. Each non-zero velocity vector $\boldsymbol{c}_q$ has its opposite $\boldsymbol{c}_{\bar{q}}$.

The population f_q can be decomposed into an even part, i.e., $f_q^+ = ½\,(f_q + f_{\bar{q}})$ and an odd part, i.e., $f_q^- = ½\,(f_q - f_{\bar{q}})$. The rule for the evolution of the populations reads:

$$f_q(\boldsymbol{r}+\boldsymbol{c}_q, t+1) = f_q(\boldsymbol{r},t) - s^+(f_q^+ - e_q^+) - s^-(f_q^- - e_q^-) \qquad \text{(S1)}$$

where s^+ and s^- are the relaxation parameters for the even and the odd part, and e_q^+ and e_q^- are the equilibrium distributions for the even and the odd part, respectively.

The relaxation parameter s^- is related to the oxygen diffusion coefficient (D, in real units) as follows:

$$s^- = \left(\frac{D\Delta t}{c_e\,\Delta x^2} + \frac{1}{2}\right)^{-1} \qquad \text{(S8)}$$

where Δx is the lattice spacing (in real units), Δt is the time step (in real units), and c_e is a scale parameter. In the D3Q7 scheme, $c_e \in]0\,;\,1/3]$. We fixed it to $c_e = 1/4$.

To ensure the optimal stability of this LB method [44], the relaxation parameter s^+ satisfied the so called "magic number relation" :

$$\left(\frac{1}{s^+} - \frac{1}{2}\right)\left(\frac{1}{s^-} - \frac{1}{2}\right) = \frac{1}{4} \qquad \text{(S9)}$$

The equilibrium distributions e_q^+ et e_q^- are given by (with Einstein notation, i.e., implicit notation over $\alpha = 1{,}3$ corresponding to the three directions x, y, and z, respectively):

$$e_q^+ = \frac{1}{2}C\left(c_e U_\alpha^2 c_{q\alpha}^2\right) + \frac{\mathcal{S}_q}{s^+}\,, \qquad e_q^- = \frac{1}{2}C U_\alpha c_{q\alpha}\,,\ \text{for } q = 1{,}6 \qquad \text{(S10)}$$

$$e_0^+ = C(\boldsymbol{r},t) - 2\sum_{q=1}^{3} e_q^+(\boldsymbol{r},t) + \frac{\mathcal{S}(\boldsymbol{r},t) - \sum_{q=1}^{6}\mathcal{S}_q}{s^+}\,, \qquad e_0^- = 0 \qquad \text{(S11)}$$

Where:

$\boldsymbol{U}(\boldsymbol{r},t) = \{U_\alpha, \alpha = 1{,}3\}$ is the macroscopic velocity in the lattice unit at the node $\boldsymbol{r}$,

$C(\boldsymbol{r},t)$ is the zeroth order moment of the particle velocity distribution given by

$$C(\boldsymbol{r},t) = \sum_{q=0}^{6} f_q(\boldsymbol{r},t) \qquad \text{(S12)}$$

$\mathcal{S}(\boldsymbol{r},t)$ is the source term (in lattice units) corresponding to the oxygen consumption by the cells and $\mathcal{S}_q = c_e\mathcal{S}/2$ is its contribution for $q = 1{,}6$.

The macroscopic oxygen concentration $c(\boldsymbol{r},t)$ (in lattice units) is computed from the zeroth order moment of the particle velocity distribution C with a correction due to the source term:

$$c(\boldsymbol{r},t) = C(\boldsymbol{r},t) + \frac{\mathcal{S}(\boldsymbol{r},t)}{2} \qquad \text{(S13)}$$

Eventually, the time step is chosen to ensure the stability of the D3Q7 LB method, i.e., $\boldsymbol{U}^2 \le \min[1-3c_e, 3c_e/2] = 1/4$ [44] which implies:

$$\Delta t \le \frac{\Delta x}{2v_{max}} \qquad \text{(S14)}$$

Where $v_{max} = \max(v)$ is the maximal local velocity in a real unit.

Simulations were run until the oxygen concentration field reached a steady state. The convergence criteria were based on the relative error on the concentration field between 1,000-time steps (expressed with the L_2 norm):

$$err^2 = \frac{\sum_{\vec{r}}\left(c(\boldsymbol{r},t) - c(\boldsymbol{r},t - 1000\,\Delta t\,)\right)^2}{\sum_{\vec{r}}\left(c(\boldsymbol{r},t)\right)^2} \quad \text{(S15)}$$

.

Supplementary Material 6: Cumulative volume-average distribution of spheroid diameters

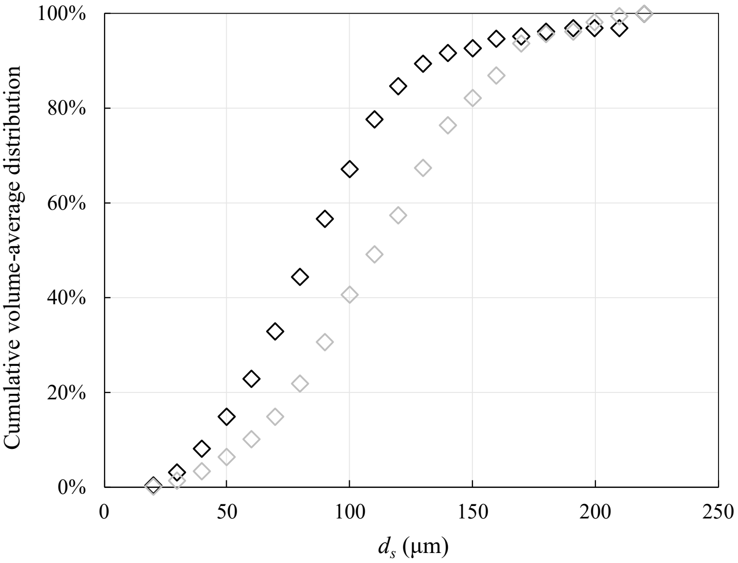

Figure S6 - Cumulative volume–average distribution of spheroid diameters

The initial cell seeding was 600,000 cells per scaffold. The dynamic conditions were run 24 hours post-seeding: scaffolds were stacked in three bioreactors (48 scaffolds per bioreactor) and perfused with a flow rate of 10 mL min^{-1}. At different time points, i.e., 24 hours post-seeding (black diamonds) and after 14 days (grey diamonds) of dynamic culture, three scaffolds were collected and analyzed by confocal microscopy (observation to 370 µm depth of entire XY cross-sections; 26 sections with a resolution of 2.5 µm). The number and the individual volume of the spheroids were calculated by image processing (ImageJ) using the cell membranes' signal of PKH-26-pre-labeled cells.

Supplementary Material 7: Mass transfer limitations according to standard correlations

We carried out some hand calculations to analyze the oxygen transport within the bioreactor. Calculations were performed for a bioreactor filled with 48 scaffolds seeded with 400,000 cells each, perfused with a flow rate $q = $ 10 mL min^{-1} with inlet oxygen concentration $c_0 = 0.21$ mol m^{-3}.

First of all, we estimated the maximal fraction of the oxygen mass flow rate entering the bioreactor taken by the cells:

$$\frac{48 \times 400{,}000 \times V_{max}}{qc_0} = 4.95\% \quad \text{(S16)}$$

In the bioreactor, the transport of dissolved oxygen from the culture medium to the cells is a three-step process, i.e., (i) convective transport in the macroporosity of the bioreactor, (ii) diffusional transport in the hydrogel scaffolds, then (iii) diffusional transport and simultaneous consumption within the spheroids.

The convective mass transfer coefficient of dissolved oxygen (k_m) can be evaluated for our bioreactor from a correlation established by Seguin et al. [84] for a similar packed bed. Seguin et al. [84] studied liquid-solid mass transfer in packed beds of variously shaped particles. The closest configuration to ours is a packed bed of plates with an aspect ratio $e/a = 0.209$ where e is the plate thickness and a is its side length, to be compared with the aspect ratio of our scaffold equal to 0.175. In this configuration, the pore Sherwood number is given by the correlation (S17):

$$\mathrm{Sh} = 1.29\ \mathrm{Re}^{1/3}\ \mathrm{Sc}^{1/3} \quad \text{(S17)}$$

The pore Sherwood number is defined by:

$$\mathrm{Sh} = \frac{4k_m\varepsilon}{D_0 A(1-\varepsilon)} \quad \text{(S18)}$$

where k_m is the convective mass transfer coefficient, D_0 the molecular diffusion of dissolved oxygen in the liquid phase, A the dynamic specific area of the packed bed (i.e., the surface area really presented to the flow), and ε is its porosity.

The pore Reynolds number Re reads:

$$\mathrm{Re} = \frac{4u_s\tau\rho}{\mu A(1-\varepsilon)} \quad \text{(S19)}$$

where τ is the tortuosity of the packed bed.

Sc is the Schmidt number given by $\mathrm{Sc} = \nu/D_0$.

A and ε were estimated from the MRI 3D image of a scaffold stack. We took $\tau = 2.77$, i.e., the value reported by Seguin et al. for the packed bed of plates with aspect ratio $e/a = 0.209$.

For our dynamic culture conditions, i.e., $u_s = 1.47$ mm s^{-1}, we found $\mathrm{Re} = 15$. The Schmidt was $\mathrm{Sc} = 1000$. We computed $\mathrm{Sh} = 32$ and $k_m = 3.5 \times 10^5$ m s^{-1}.

The Biot number denoted Bi, allows comparing the diffusion mass transfer resistance in the hydrogel to the convective mass transfer resistance in the perfusion liquid. We chose the mean spacing between spheroids within a scaffold as the characteristic length entering the Biot number. We calculated the mean spacing between spheroids, i.e., $\ell = 400$ μm, knowing the volume of a scaffold, the number of cells per scaffold, i.e., 400,000 cells, and the spheroid diameter, i.e., 135 μm, in our bioreactor simulations. We estimated $\mathrm{Bi} = k_m\ell/D = 15$ (Bi is even greater when the scaffold half-thickness or its radius is taken as characteristic length) and deduced that the diffusion in the hydrogel is the mass transfer-controlling step.

Since $\mathrm{Bi} \gg 1$ and the oxygen consumption rate by the spheroids represented at the most 5% of the oxygen mass flow rate entering the bioreactor, it appears reasonable to assume that the oxygen concentration of the liquid phase within the macropores is approximately homogeneous and equal to c_0, the oxygen concentration at the bioreactor inlet.

The second Damkhöler number compares the oxygen consumption rate by the cells of a spheroid to the diffusive mass transfer rate of oxygen in the hydrogel:

$$\mathrm{Da} = (V_{max} d_s/\vartheta)/(Dc_0/\ell) = 4.5 \quad \text{(S20)}$$

We chose the spheroid spacing ℓ as the diffusion characteristic length (Da would be higher if the scaffold half-thickness or its radius is taken as characteristic length). Low concentration gradients within hydrogel would require $\mathrm{Da} \ll 1$. When $\mathrm{Da} \gg 1$, the diffusion step may strongly limit the oxygen consumption by the cells. The present value of the Damkhöler number lies in between.

The bioreactor simulations (400,000 cells per scaffold) showed that even if the dissolved oxygen concentration significantly varied within the hydrogel scaffolds, the spheroids did not suffer from hypoxia.

However, in some of our simulations at the scaffold scale, larger spheroids (400 μm in diameter) and/or higher cell concentration (900,000 cells per scaffold) led to hypoxic spheroids.
